# Development of Copper Complexes with Diimines and Dipicolinate as Anticancer Cytotoxic Agents

**DOI:** 10.3390/pharmaceutics15051345

**Published:** 2023-04-27

**Authors:** Natalia Alvarez, Analu Rocha, Victoria Collazo, Javier Ellena, Antonio J. Costa-Filho, Alzir A. Batista, Gianella Facchin

**Affiliations:** 1 Química Inorgánica, Departamento Estrella Campos, Facultad de Química, Universidad de la República, Montevideo 11800, Uruguay; 2 Departamento de Química, Federal University of São Carlos, CP 676, São Carlos 13565-905, SP, Brazil; 3 São Carlos Institute of Physics, University of São Paulo, Av. do Trabalhador São-Carlense 400, São Carlos 13566-590, SP, Brazil; 4 RIbeirão Preto School of Philosophy, Science and Literature, University of São Paulo, Av. Bandeirantes, Ribeirão Preto 14040-901, SP, Brazil

**Keywords:** copper complexes, dipicolinic acid, phenanthroline, 4-methyl-1,10-phenanthroline, neocuproine, 4,7-diphenyl-1,10-phenanthroline, 2,2′-dipirydil-amine, bipyridine, DNA binding, cytotoxic activity

## Abstract

Coordination complexes may act as anticancer agents. Among others, the formation of the complex may facilitate the ligand uptake by the cell. Searching for new copper compounds with cytotoxic activity, the complex Cu-dipicolinate was studied as a neutral scaffold to form ternary complexes with diimines. A series of [Cu(dipicolinate)(diimine)] complexes (where diimine: Phenanthroline, phen, 5-NO_2_-phenanthroline, 4-methyl-phenanthroline, neocuproine, 3,4,7,8-tetramethyl-phenanthroline, tmp, bathophenanthroline, bipyridine, dimethyl-bipyridine, as well as the ligand 2,2-dipyridil-amine, bam) were synthesized and characterized both in the solid state, including a new crystal structure of [Cu_2_(dipicolinate)_2_(tmp)_2_]·7H_2_O. Their chemistry in aqueous solution was explored by UV/vis spectroscopy, conductivity, cyclic voltammetry, and electron paramagnetic resonance studies. Their DNA binding was analyzed by electronic spectroscopy (determining *K_b_* values), circular dichroism, and viscosity methods. The cytotoxicity of the complexes was assessed on human cancer cell lines MDA-MB-231, MCF-7 (breast, the first triple negative), A549 (lung epithelial) and A2780cis (ovarian, Cisplatin-resistant), and non-tumor cell lines MRC-5 (lung) and MCF-10A (breast). The major species are ternary, in solution and solid state. Complexes are highly cytotoxic as compared to Cisplatin. Complexes containing bam and phen are interesting candidates to study their in vivo activity in triple-negative breast cancer treatment.

## 1. Introduction

Cancer causes about 19 million new cases and 10 million deaths yearly (worldwide data for 2020) [1], evidencing the need for new drugs to treat this disease. Platinum coordination compounds play an important role in its treatment. Cisplatin and congeners (carboplatin, oxaliplatin, heptaplatin, and picoplatin) are successfully used against various tumor classes, even as part of curative treatments [2,3].

Developing new metal coordination compounds (“complexes”) with potential anticancer activity offers a broad range of possibilities, from varying the metal centers and ligands to combining their properties, that could lead to new metallo-pharmaceuticals. Several metal centers have been identified to give rise to complexes that present antitumor activity, such as Pt, Ru, Ga, Au, and Cu, among others [3,4]. This research has remained mainly in academia, possibly due to the complex chemical reactivity and the variety of mechanisms of action that coordination compounds may present [5].

**Figure 1:**
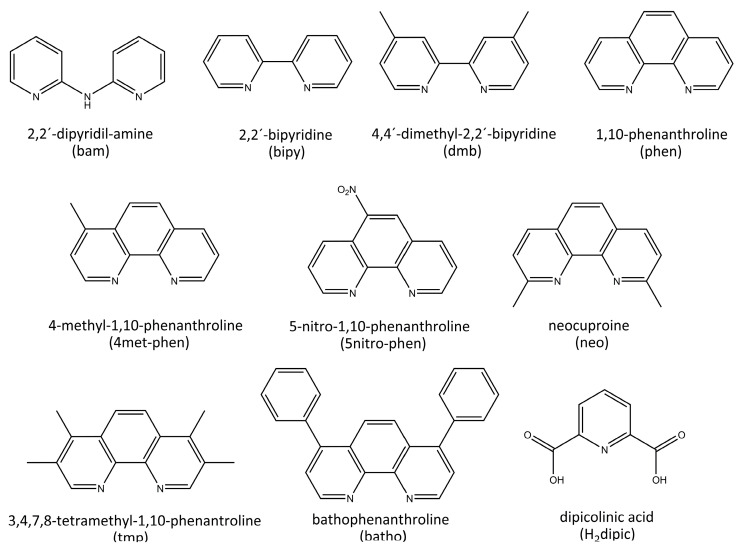
Chemical structures of the ligands used in this work.

Diimines, such as bipyridine (bipy) and phenanthroline (phen) derivatives (Figure 1), exhibit antineoplastic activity. Diiminic ligands are extensively utilized in medicinal inorganic chemistry, mainly searching for new antitumor compounds. It has been demonstrated that phen inhibited DNA synthesis in cells at micromolar concentrations [6].

Differentiating the biological effects of metals versus their coordinated ligands can be difficult in coordination compounds. A current issue is whether the cytotoxic activity of diimine coordination compounds results from the intact complex, i.e., the metal bound to the organic molecule (the ligand), or if the metal transfers the ligand into the cell, acting as a delivery moiety. Both phenomena seem to coexist in the present-day state of knowledge [6]. The metal complexes have been demonstrated to modify cellular uptake, but subsequent ligand exchange and reactions with endogenous intracellular metals and ligands may be implicated in the biological activity. For instance, studies with radiolabelled neocuproine (neo) show that the treatment with the Cu-neo complex increases 200-fold the neo concentration inside the cell compared to the treatment with only free, not complexed, neo [7].

Our research group is devoted to finding new copper compounds with cytotoxic activity that may lead to anticancer agents. We used different Cu-ligand scaffolds to incorporate the diimine as a ligand to facilitate cellular uptake and enhance activity. To date, our research was mainly centered on the Cu-dipeptide scaffold. Different series of [Cu(L-dipeptide)(diimine)] compounds were developed (where diimine: Phen, 5-NO_2_-phenanthroline, 5nitro-phen, neocuproine, neo, 3,4,7,8-tetramethyl-phenanthroline, tmp, bathophenanthroline, batho and L-dipeptide: Ala-Gly, Ala-Phe, Phe-Ala, and Phe-Phe among others) [8,9,10,11,12]. The obtained compounds constitute potent cytotoxic agents, more active than Cisplatin, with neo- and tmp-containing complexes being the most active of the group (with IC_50_ in the low micromolar or sub-micromolar range).

Aiming at identifying compounds with improved activity against cancer cells, we studied other neutral Cu-ligand scaffolds for diimine incorporation to ascertain their influence on cytotoxic activity [13]. In this work, we present the study of the Cu-dipicolinate system as a scaffold to bind diimines and other ligands that may lead to cytotoxic compounds (where diimine: Bipyridine, bipy and dimethyl-bipyridine, dmb, 4-methyl-phenanthroline, 4met-phen, 5nitro-phen, neo, batho, tmp, as well as the ligand 2,2′-dipirydil-amine, bam). Dipicolinate (dipic) forms stable compounds with copper, which allows the insertion of other bidentate ligands, making stable ternary complexes (i.e., including two different ligands in the coordination sphere of the metal) [14]. The complexes were characterized both in solid state and in aqueous solution, including a new XRD structure for the complex [Cu_2_(dipic)_2_(tmp)_2_]·7H_2_O. As many metallic complexes bind to DNA as a part of their mechanism of action, its interaction with isolated Calf Thymus-DNA (CT-DNA) was studied by circular dichroism (CD), electronic spectroscopy (determining binding constants, *K_b_*), and viscosity methods. The cytotoxicity of the complexes was assessed against MDA-MB-231, MCF-7 (human metastatic breast adenocarcinomas, the first triple negative), MCF-10A (human non-tumoral breast cells), A549 (human lung epithelial carcinoma), MRC-5 (human non-tumoral lung epithelial cells), and A2780cis (Cisplatin-resistant human ovarian carcinoma).

## 2. Materials and Methods

All chemical compounds (Sigma-Aldrich, St. Louis, MI, USA) and solvents were used as commercially available without further purification.

### 2.1. Synthetic Procedure

To a suspension of dipicolinic acid (0.15 mmol) in 10 mL of water basic copper(II) carbonate was added in 100% excess and allowed to react with constant stirring at 60 °C for 30 min [15]. The mixture was filtered off, and a limpid blue solution was obtained, which contained the Cu-dipic complex. To this solution, 5 mL of a 30 mM solution of the ligand (diimine or bam), was added with constant stirring for 15 min at 60 °C. A green-blue solution was obtained and left to evaporate at room temperature or at 4 °C slowly until blue crystals were obtained. The complexes were obtained by the following equations.

$$\mathrm{Cu}\left({\mathrm{CO}}_{3}\right)\mathrm{Cu}{\left(\mathrm{OH}\right)}_{2}\left(s\right)+2H_{2}\mathrm{dipic}\left(s\right)\overset{H_{2}O,\;}{\to }2\left[\mathrm{Cu}\left(\mathrm{dipic}\right){\left(H_{2}O\right)}_{2}\right]\left(\mathrm{aq}\right)+{\mathrm{CO}}_{2}\left(g\right)$$

$$\left[\mathrm{Cu}\left(\mathrm{dipic}\right){\left(H_{2}O\right)}_{2}\right]\left(\mathrm{aq}\right)+\;\mathrm{NN}\left(\mathrm{aq}\right)\overset{H_{2}O:\mathrm{EtOH}\;2:1,\;}{\to }2\left[\mathrm{Cu}\left(\mathrm{dipic}\right)\left(\mathrm{NN}\right){\left(H_{2}O\right)}_{x}\right]\left(\mathrm{ac}\right)+\left(2-x\right)H_{2}O\left(l\right)$$

Nine ternary complexes containing dipic were obtained. Single crystals appropriate for X-ray diffraction studies were obtained for seven of them.

- [Cu(dipic)(bam)]·3H_2_O. (**1**) Green solid. Yield 76%. Elemental analysis Calc. for CuC_17_H_18_N_4_O_7_/Exp. %C 44.98/44.80, %N 12.34/12.38, %H 4.00/3.52. Purity 99%. FT-IR: 2900–3500 cm^−1^ ν(C-H), ν(O-H), ν(N-H); 1621 cm^−1^ ν(COO^−^)_as_; 1537 cm^−1^ ν(C-C), ν(C-N); 1433 cm-1 ν(C-C), ν(C-N), ν(COO^−^)_s_; 1163 and 1079 cm^−1^ ρ(C-H); 912 cm^−1^ δ(C-C-C), δ(C-C-N); 879 cm^−1^ δ_oop_(C-H), δ(C-C-C), δ(C-C-N); 730 cm^−1^ ρ(C-H); 538 and 443 cm^−1^ ν(Cu-N/O). Λ_M_ (25 °C): 28 Scm^2^ mol^−1^.
- [Cu_2_(dipic)_2_(bipy)_2_]·12H_2_O. (**2**) Blue solid. Yield 32%. Elemental analysis Calc. for CuC_17_H_23_N_3_O_10_/Exp. %C 41.42/41.79, %N 8.52/8.37, %H 4.70/4.29. Purity 98%. FT-IR: 2900–3500 cm^−1^ ν(C-H), ν(O-H), ν(N-H); 1631 cm^−1^ ν(COO^−^)_as_; 1528 cm^−1^ ν(C-C), ν(C-N); 1446 cm-1 ν(C-C), ν(C-N), ν(COO^−^)_s_; 1159 and 1088 cm^−1^ ρ(C-H); 910 cm^−1^ δ(C-C-C), δ(C-C-N); 857 cm^−1^ δ_oop_(C-H), δ(C-C-C), δ(C-C-N); 731 cm^−1^ ρ(C-H); 537 and 414 cm^−1^ ν(Cu-N/O). Λ_M_ (25 °C): 15 Scm^2^ mol^−1^.
- [Cu(dipic)(dmb)]·7H_2_O. (**3**) Blue solid. Yield 24%. Elemental analysis Calc. for CuC_19_H_29_N_3_O_11_/Exp. %C 42.34/41.80, %N 7.80/7.71, %H 5.42/5.22. Purity 98%. FT-IR: 2900–3500 cm^−1^ ν(C-H), ν(O-H), ν(N-H); 1616 cm^−1^ ν(COO^−^)_as_; 1492 cm^−1^ ν(C-C), ν(C-N); 1427 cm-1 ν(C-C), ν(C-N), ν(COO^−^)_s_; 1151 and 1084 cm^−1^ ρ(C-H); 910 cm^−1^ δ(C-C-C), δ(C-C-N); 842 cm^−1^ δ_oop_(C-H), δ(C-C-C), δ(C-C-N); 724 cm^−1^ ρ(C-H); 557 and 448 cm^−1^ ν(Cu-N/O). Λ_M_ (25 °C): 8 Scm^2^ mol^−1^.
- [Cu(dipic)(phen)(H_2_O)]·2H_2_O. (**4**) Blue solid. Yield 31%. Elemental analysis Calc. for CuC_19_H_17_N_3_O_7_/Exp. %C 49.30/49.73, %N 9.08/9.33, %H 3.70/3.44. Purity 97%. FT-IR: 2900–3500 cm^−1^ ν(C-H), ν(O-H), ν(N-H); 1624 cm^−1^ ν(COO^−^)_as_; 1520 cm^−1^ ν(C-C), ν(C-N); 1427 cm-1 ν(C-C), ν(C-N), ν(COO^−^)_s_; 1145 and 1083 cm^−1^ ρ(C-H); 910 cm^−1^ δ(C-C-C), δ(C-C-N); 851 cm^−1^ δ_oop_(C-H), δ(C-C-C), δ(C-C-N); 723 cm^−1^ ρ(C-H); 544 and 442 cm^−1^ ν(Cu-N/O). Λ_M_ (25 °C): 16 Scm^2^ mol^−1^.
- [Cu(dipic)(4met-phen)]·2.5H_2_O. (**5**) Blue solid. Yield 51%. Elemental analysis Calc. for CuC_20_H_18_N_3_O_6.5_/Exp. %C 51.34/51.02, %N 8.98/8.91, %H 3.88/4.04. Purity 99%. FT-IR: 2900–3500 cm^−1^ ν(C-H), ν(O-H), ν(N-H); 1627 cm^−1^ ν(COO^−^)_as_; 1523 cm^−1^ ν(C-C), ν(C-N); 1427 cm-1 ν(C-C), ν(C-N), ν(COO^−^)_s_; 1188 and 1087 cm^−1^ ρ(C-H); 913 cm^−1^ δ(C-C-C), δ(C-C-N); 859 cm^−1^ δ_oop_(C-H), δ(C-C-C), δ(C-C-N); 727 cm^−1^ ρ(C-H); 541 cm^−1^ ν(Cu-N/O). Λ_M_ (25 °C): 19 Scm^2^ mol^−1^.
- [Cu(dipic)(5nitro-phen)(H_2_O)]·7.5H_2_O. (**6**) Blue solid. Yield 43%. Elemental analysis Calc. for CuC_19_H_27_N_4_O_14.5_/Exp. %C 37.60/37.75, %N 9.23/9.28, %H 4.48/4.15. Purity 99%. FT-IR: 2900–3500 cm^−1^ ν(C-H), ν(O-H), ν(N-H); 1622 cm^−1^ ν(COO^−^)_as_; 1521 cm^−1^ ν(C-C), ν(C-N); 1427 cm-1 ν(C-C), ν(C-N), ν(COO^−^)_s_; 1185 and 1087 cm^−1^ ρ(C-H); 918 cm^−1^ δ(C-C-C), δ(C-C-N); 856 cm^−1^ δ_oop_(C-H), δ(C-C-C), δ(C-C-N); 735 cm^−1^ ρ(C-H); 519 and 443 cm^−1^ ν(Cu-N/O). Λ_M_ (25 °C): 11 Scm^2^ mol^−1^.
- [Cu(dipic)(neo)]·3H_2_O. (**7**) Blue solid. Yield 55%. Elemental analysis Calc. for CuC_21_H_20_N_3_O_6.5_/Exp. %C 52.34/52.22, %N 8.72/8.75, %H 4.18/3.67. Purity 99%. FT-IR: 2900–3500 cm^−1^ ν(C-H), ν(O-H), ν(N-H); 1635 cm^−1^ ν(COO^−^)_as_; 1503 cm^−1^ ν(C-C), ν(C-N); 1424 cm-1 ν(C-C), ν(C-N), ν(COO^−^)_s_; 1146 and 1090 cm^−1^ ρ(C-H); 915 cm^−1^ δ(C-C-C), δ(C-C-N); 853 cm^−1^ δ_oop_(C-H), δ(C-C-C), δ(C-C-N); 741 cm^−1^ ρ(C-H); 547 and 434 cm^−1^ ν(Cu-N/O). Λ_M_ (25 °C): 13 Scm^2^ mol^−1^.
- [Cu(dipic)(batho)(H_2_O)]·H_2_O. (**8**) Blue solid. Yield 37%. Elemental analysis Calc. for CuC_31_H_23_N_3_O_6_/Exp. %C 62.36/62.58, %N 7.04/7.12, %H 3.88/3.49. Purity 98%. FT-IR: 2900–3500 cm^−1^ ν(C-H), ν(O-H), ν(N-H); 1624 cm^−1^ ν(COO^−^)_as_; 1495 cm^−1^ ν(C-C), ν(C-N); 1427 cm-1 ν(C-C), ν(C-N), ν(COO^−^)_s_; 1152 and 1089 cm^−1^ ρ(C-H); 913 cm^−1^ δ(C-C-C), δ(C-C-N); 851 cm^−1^ δ_oop_(C-H), δ(C-C-C), δ(C-C-N); 738 cm^−1^ ρ(C-H); 549 and 443 cm^−1^ ν(Cu-N/O). Λ_M_ (25 °C): 13 Scm^2^ mol^−1^.
- [Cu_2_(dipic)_2_(tmp)_2_]·7H_2_O. (**9**) Blue solid. Yield 28%. Elemental analysis Calc. for CuC_23_H_26_N_3_O_7.5_/Exp. %C 52.32/52.26, %N 7.95/7.83, %H 4.96/4.58. Purity 98%.FT-IR: 2900–3500 cm^−1^ ν(C-H), ν(O-H), ν(N-H); 1621 cm^−1^ ν(COO^−^)_as_; 1528 cm^−1^ ν(C-C), ν(C-N); 1425 cm-1 ν(C-C), ν(C-N), ν(COO^−^)_s_; 1157 and 1081 cm^−1^ ρ(C-H); 910 cm^−1^ δ(C-C-C), δ(C-C-N); 870 cm^−1^ δ_oop_(C-H), δ(C-C-C), δ(C-C-N); 724 cm^−1^ ρ(C-H); 552 and 443 cm^−1^ ν(Cu-N/O). Λ_M_ (25 °C): 10 Scm^2^ mol^−1^.

Infrared spectra for all complexes are available in Figure S1 in the Supplementary Information.

### 2.2. Analytical and Spectroscopic Characterization

Chemical analyses of carbon, nitrogen, and hydrogen were carried out in a Thermo Scientific Flash 2000 analyzer.

FT-IR spectra were obtained using KBr in the 4000 to 400 cm^−1^ region on a Shimadzu IR Prestige 21 spectrometer. UV–visible (UV–vis) spectra of aqueous solutions of the complexes were recorded on a Thermo Scientific Evolution 60 spectrophotometer in a 1 cm-path length quartz cells.

Conductivity measurements of 1 mM aqueous solutions of the complexes were performed in a Jenway Conductivity Meter 4310, at 25 °C.

EPR measurements of aqueous solutions at room temperature were carried out at X-band (9.5 GHz) using a JEOL JES-FA200 spectrometer and a cavity with 100 kHz field modulation. Other experimental parameters were optimized to maximize the signal-to-noise ratio and avoid spectra saturation.

Cyclic voltammetry studies were performed in 1 mM aqueous solutions of the complexes (**2**), (**3**), (**4**), and (**5**) in 0.1 M KCl, in a Dropsens µStat potentiostat (Metrohm) with a disposable carbon electrode (DRP-110, Metrohm). Potassium ferrocyanide was used as internal reference. Voltammograms were obtained at 0.05, 0.10, 0.150, and 200 mV/s to verify reversibility for the Cu(II)/Cu(I) process.

### 2.3. Thermal Stability Analysis

Thermogravimetric analysis of the solid samples was performed on a Shimadzu TGA-50 in the 25–800 °C range using alumina cells with 50 mg/L N_2_ flow. Differential scanning calorimetry was measured in the 25–600 °C range in aluminum cells with 50 mg/L N_2_ flow on a Shimadzu DSC-60.

### 2.4. Crystal Structure Determination and Analysis

Single crystals of complex (**9**) were studied on a Bruker D8 Venture diffractometer, using graphite monochromated MoKα radiation (0.71073 Å) at 298(2). Data treatment was performed using APEX2 software (Madison, MI, USA) [16,17]. Multi-scan absorption correction was applied using SADABS (Madison, MI, USA) [18]. Intrinsic phasing methods were used to solve the structure in SHELXT. Full-matrix least-squares on F^2^ refinement was carried out in SHELXL-2018/3 [19] within Olex2 [20]. All non-hydrogen atoms were refined anisotropically and H atoms were treated according to the *riding* model. Due to highly disordered water molecules in the structure, a solvent mask [21] was applied, yielding 20 water molecules per unit cell, corresponding to 5 water molecules per dinuclear copper(II) complex. Details are embedded in the CIF file. The presented empirical formula in Table 1 corresponds to the element summation without considering the solvent mask.

Structure visualization and image preparation were performed on Mercury [22]. Crystallographic data and refinement results are listed in Table 1.

The corresponding CIF file was deposited in the Cambridge Structural Database (deposition number 2241020). Copies are available free of charge through the access structures applet in the CCDC webpage (https://www.ccdc.cam.ac.uk/structures/).

In order to further explore the influence of the intermolecular interactions in the crystal structure on relevant physicochemical parameters, such as lipophilicity a complete analysis of the Hirshfeld surface was conducted in CrystalExplorer21 [23]. 2D fingerprint plots and the normalized atom distance to and from the mapped surface (*d*_norm_) were analized, where *d*_norm_ is: $d_{norm}=\frac{d_{i}-r_{i}^{vdW}}{r_{i}^{vdW}}+\frac{d_{e}-r_{e}^{vdW}}{r_{e}^{vdW}}$, *d*_e_ and *d*_i_ represent the distances from a point on the surface to the nearest nucleus outside (*d*_e_) and inside (*d*_i_) the surface, respectively, and *r*^vdW^ corresponds to the van der Waals (vdW) radii of the atoms involved. 2D fingerprint plots represent a map of *d*_e_ vs. *d*_i_ distances within the surface (and their frequency), providing quantitative information on the type of intermolecular contacts in the lattice [24]. This analysis was based on the deposited CIF files on the CSD for complexes (**1**), (**4**), (**6**), and (**8**) (Refcodes: NOHCET, VELNOQ, WAWKAH, and CAXBIN, respectively).

### 2.5. Lipophilicity and DNA Interaction Studies

The lipophilicity of the complexes was studied by Thin Layer Reverse Chromatography using ALUGRAM^®^ RP-18W/UV254 plates. DMSO solutions of the complexes were applied and then dried for 12 h at 50 °C. A methanol:water 9:1 mixture in the presence of Tris/HCl pH = 7.4 5 mM buffer was used for elution. Results are reported as R_M_ values obtained from the determined R_f_ using the expression R_M_ = log10[(1/R_f_) − 1] [25].

Absorption titration measurements in the UV were carried out keeping the complex concentration constant at 10–15 µM in 5 mM buffer Tris/HCl pH = 7.5 and 50 mM of NaCl while varying the concentration of Calf Thymus-DNA (CT-DNA) from 0 to 250 μM. The intrinsic binding constants (K_b_) were determined using the Benesi–Hildebrand method [26] by calculating the ratio of the slope to the intercept of the [complex]/A_obs_ as a function of 1/[DNA] plot.

Circular dichroism spectra of aqueous solutions of DNA in the presence of increasing amounts of copper complexes were recorded on a JASCO J-815 equipment in a 1 cm path-length quartz cells. The scan range was 220–320 nm with a scanning rate of 100 nm/min, a response time of 1 s, and at least 4 accumulations. The DNA concentration was kept constant at approximately 10 µM in 5 mM Tris/HCl pH = 7.4/50 mM NaCl, and the complex concentration was varied to obtain spectra with [DNA]/[complex] ratio between 20 and 1.

Viscosity measurements were performed in an Ostwald-type viscosimeter maintained at a constant temperature of 25.0 ± 0.1 °C in a thermostatic bath. Solutions of Calf thymus-DNA (CT-DNA, 150 µM b.p.) and complexes were prepared separately in Tris-HCl (5 mM, pH = 7.2, 50 mM NaCl) and thermostatized at 25 °C. Complex−DNA solutions (6 mL) were prepared just prior to running each experiment at different molar ratios ([complex]/[CT-DNA] = 0.025, 0.050, 0.075, 0.100, and 0.125, equivalent to [DNA]/[complex] ratio contained values of 40, 20, 13, 10, and 8). Solutions were equilibrated for 15 min at 25 °C and then 5 flow times were registered. The relative viscosity of DNA in the absence (η_0_) and presence (η) of complexes was calculated as: (η/η_0_) = t − t_0_/t_DNA_ − t_0_, where t_0_ and t_DNA_ are the flow times of the buffer and DNA solution, respectively, while t is the flow time of the DNA solution in the presence of copper complexes. Data are presented as (η/η^0^)^1/3^ versus the ratio [complex]/[DNA] [27].

### 2.6. Cytotoxicity Studies

The cytotoxicity of the complexes was evaluated against different human cancer cell lines: Human metastatic breast adenocarcinoma MDA-MB-231 (triple negative, ATCC: HTB-26), MCF-7 (hormone-dependent ATCC: HTB-22), human lung epithelial carcinoma A549 (ATCC: CCL-185), A2780cis (Cisplatin-resistant human ovarian carcinoma, SIGMA), and non-tumoral cell lines: MRC-5 (lung; ATCC: CCL-171) and MCF-10A (breast, ATCC: CRL-10317). The 3-(4,5-dimethylthiazol-2-yl)-2,5-diphenyltetrazolium bromide (MTT) colorimetric assay was used. The cells were cultured in Dulbecco’s Modified Eagle’s Medium (DMEM) for MDA-MB-231, A549, and MRC-5, supplemented with 10% fetal bovine serum (FBS), Roswell Park Memorial Institute (RPMI) 1640 Medium for MCF-7 and A2780cis supplemented with 10% FBS, or Dulbecco’s Modified Eagle Medium Nutrient Mixture F-12 (DMEM F-12) for MCF-10A, containing 5% horse serum, Epidermal growth factor (EGF, 20 ng/mL), hydrocortisone (0.5 µg/mL), insulin (0.01 mg/mL), 1% penicillin, and 1% streptomycin, at 310 K in humidified 5% CO_2_ atmosphere. In the assay, 1.5 × 10^4^ cells/well were seeded in 150 µL of the medium in 96-well plates and incubated at 37 °C in 5% CO_2_ for 24 h, to allow cell adhesion. The cells were then treated with the copper complexes for 48 h. The complexes were previously dissolved in DMSO (0.5% *v*/*v*), and 0.75 µL of solution was added to each well with 150 µL of the medium. Cisplatin, as a DMF solution, was used as a reference drug. MTT (50 µL, 1 mg/mL in PBS) was added to each well after the treatment, and the plate was further incubated for 3 h. Cell viability was detected by the reduction of MTT to purple crystals of formazan by living cells, which were then solubilized by isopropanol (150 µL/well). The optical density of each well was measured at 540 nm. The concentration that reduced cell viability to 50% (IC_50_) was obtained from three independent experiments via the analysis of absorbance data on GraphPad Prism 8 (Graph Pad Software, San Diego, CA, USA).

## 3. Results and Discussion

Nine ternary copper(II) coordination compounds with general formula [Cu(dipic)(NN)(H_2_O)_x_]·yH_2_O were obtained (NN = bam, bipy, dmb, phen, 4met-phen, 5nitro-phen, neo, batho and tmp, x = 0–1 and y = 0–12 depending on the NN ligand). Among those, six yielded single crystals suitable for X-ray diffraction studies. Five of the obtained crystals were tested and corresponded to previously reported crystal structures, complexes (**1**) [28], (**2**) [29], (**4**) [30], (**7**) [31], and (**8**) [32]. To the best of our knowledge, no further studies related to biological activity were performed for those compounds. Complexes (**3**), (**5**), (**6**), and (**9**) are described for the first time.

Table 2 summarizes the characteristics of the copper environment in the complexes, including coordination index, geometry, and equatorial and axial donors.

The complexes present five or six coordination indexes where the position of the donor atoms varies depending on the geometry of the metal center. The dipic is situated in the equatorial plane for five coordinated compounds, and the diimine is approximately perpendicular to it, as previously reported for [Cu(dipeptide)(phen)] complexes [8,9,10,11,12]. On the other hand, in six-coordinated mononuclear moieties, the diimine lies in the equatorial plane and the dipic perpendicular to it. The fourth coordinating position of the equatorial plane is completed with a water molecule. In dinuclear [Cu_2_(dipic)_2_(bipy)_2_]·12H_2_O and [Cu_2_(dipic)_2_(tmp)_2_]·7H_2_O a carboxylic oxygen atom from the dipic, coordinated in the equatorial plane, acts as a bridge between the two metal centers.

### 3.1. [Cu_2_(dipic)_2_(tmp)_2_]·7H_2_O: Crystal Structure

**Figure 2:**
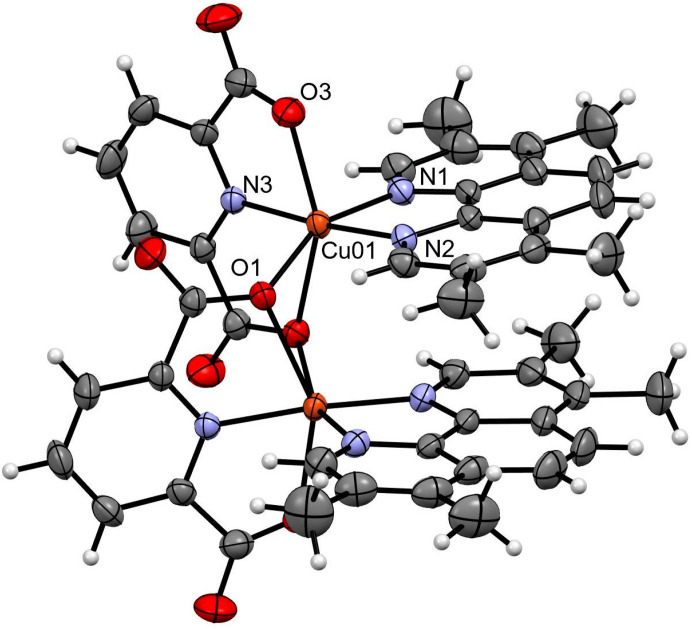
Fifty percent probability Ortep representation of the [Cu_2_(dipic)_2_(tmp)_2_] molecular moiety. Water molecules are omitted for clarity. Color code: Cu (orange), O (red), N (lilac), C (grey), H (white).

The complex containing tmp (**9**) is a dinuclear one. Each copper(II) center presents an octahedral coordination geometry with tetragonal distortion, and the dipic acts as a tridentated ditopic ligand connecting both metal centers (Figure 2). Due to a second-order rotation axis in the molecule, the asymmetric unit presents a symmetrically independent half molecule (Figure S2 and S3, Supplementary Information).

Bond lengths and angles around the copper(II) center are presented in Table 3. Distances Cu-N within the equatorial plane are around 2.0 Å. Oxygen atoms from the dipic occupy axial positions, with Cu-O distances in the 2.2–2.5 Å range.

The Cu-Cu distance is 3.317 Å. The dinuclear moiety is further stabilized through intramolecular π···π interactions where the tmp molecules are parallel to each other with inter-centroid distances of 3.753 Å (Figure S4, Supplementary Information).

To assess the intermolecular interactions in which complex (**9**) can participate, a Full Interaction Map (FIM) was constructed using Mercury (Figure S5a, Supplementary Information). This map depicts potential H-bond donor-acceptor zones surrounding the carboxylate groups in the dipic molecule as well as π stacking propensity above and below the tmp molecule. When superposing adjacent molecules, it can be observed that the potential interactions determined from the FIM are the ones that dominate the packing (Figure S5b, Supplementary Information).

**Figure 3:**
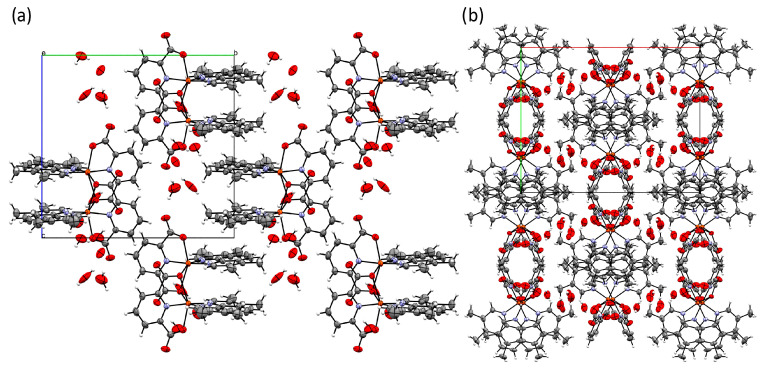
Crystal packing arrangement, view along *a* (**a**), view along *c* (**b**). Color code: Cu (orange), O (red), N (lilac), C (grey), H (white).

Figure 3 depicts the crystal packing arrangement. Supramolecular infinite chains along the *c* axis are formed through the intercalation of dinuclear units sustained by intermolecular π···π interactions, with the tmp molecules of contiguous dinuclear units parallel to each other and inter-centroid distances of 3.780 Å. These chains are connected by classical O-H···O and non-classical C-H···O hydrogen bonds involving the dipic and the hydration water molecules in the lattice. H-bond distances are in the 2.1–3.1 Å range.

### 3.2. Thermal Stability and Coordination Sphere Verification

TGA and DSC measurements were carried out for complexes of the [Cu(dipic)(NN)(H_2_O)_x_] family to verify in the bulk samples the number of solvent molecules that are hydration or coordinated, and, therefore, the degrees of hydration. This allowed us to assess which compounds are hexacoordinated in the solid state, including complexes (**5**) and (**6**), where no crystal structure is available.

In general, a first dissolution step is observed up to 200 °C, followed by the loss of a coordinated molecule in the hexacoordinated complexes between 200 and 250 °C. The studied samples are thermally stable until at least 170 °C. Complexes (**1**), (**2**), (**3**), and (**4**) melt around 250 °C before decomposition. The other complexes decompose directly from the solid state. The final solid residues are different depending on the diiminic ligand. This behavior in nitrogen flow has been previously observed for Zn-dipicolinate complexes [33]. In all cases, the decomposition starts by losing the diiminic ligand and simultaneous or subsequent loss of the dipic unit. In particular, some reports show that [Cu(dipic)] does not normally decompose entirely in a nitrogen atmosphere even if high temperatures are achieved [34] (800 °C in our conditions). Details showing mass loss percentages, associated enthalpies for water loss, and temperatures are available in Table S1 in the Supplementary Information.

In the case of complexes where the crystal structure was determined, TGA results are consistent with what is expected from the structure, confirming that the bulk’s identity is the same as in crystals. As for complexes (**5**) and (**6**), the proposed formulas are: [Cu(dipic)(4metil-phen)]·2.5H_2_O and [Cu(dipic)(5nitro-phen)(H_2_O)]·2H_2_O, respectively, confirming 5 and 6 coordinated copper(II) centers.

### 3.3. Aqueous Solution Characterization

As the ligands usually are labile in copper complexes and, therefore, can be exchanged rapidly upon dissolution, different studies were performed to assess the coordination in solution. Molar conductivities for the complexes’ present values between 8 and 30 Scm^2^ mol^−1^, suggesting that the major species in solution is the ternary, neutral one. EPR spectra in aqueous solutions at room temperature of the complexes that were dinuclear in the solid state, complexes (**2**) and (**9**) (Figure S6, Supplementary Information) are characteristic of isolated mononuclear discrete copper(II) complexes, so their major species in solution are monomeric [35,36,37].

Further information about the coordination of the ligands in the species in solution can be obtained by analyzing the visible spectra of the complexes using the empirical correlation between the visible spectrum λ_max_ and the donor atoms coordinated equatorially to the Cu(II) [38,39]. The λ_max_ of the visible spectra for the proposed equatorial donors of the penta- and hexa-coordinated complexes in the solid state calculated according to Prenesti et al. [39,40] are 641 and 632 nm, respectively. However, a bathochromic shift is expected due to axial donors of about 31–45 nm for one pyridinic N atom in the axial position and 19–44 nm for each carboxylate O [40]. In summary, the expected λ_max_ values, if the coordination scheme in the solid state was maintained in solution, are 672–686 and 670–720 nm for penta- and hexa-coordinated complexes, respectively.

Electronic spectra were measured in DMSO and aqueous solutions (Figure S7 Supplementary Information). Table 4 presents a summary of the obtained results. Considering that the experimental λ_max_ for all complexes is higher than 700 nm, there is a marked preference for hexacoordination in solution where the diiminic ligand sits in the equatorial plane, and the dipicolinate moiety rests perpendicular to it. The sixth position, in most cases, is occupied by a solvent molecule, evidenced by the fact that DMSO λ_max_ is higher than that of aqueous solution. This has been previously reported for the [Cu(dipic)(bam)] complex measured in DMF where λ_max_ is 760 nm [28]. For those complexes where λ_max_ does not vary significantly, a pentacoordinated moiety with a trigonal bipyramid geometry is expected to be the major species. In conclusion, compounds (**1**), (**2**), (**3**), (**6**), and (**8**) present a six-coordination sphere, whereas (**4**), (**5**), and (**7**) a five-coordination one in solution.

The E°´_1/2_ values of the Cu(II)/Cu(I) redox couple in aqueous solution are between those reported for Cu-dipic (−278 mV) and Cu-diimine complexes (Table 4, Figure S14 Supplementary Materials) [41], in agreement with the complexes being ternary in solution, as previously discussed. Complexes (**2**) and (**3**) containing bipy and dmp present lower values than the phen-containing complexes (**4**) and (**5**), due to the different electron density on the copper atom induced by the diiminc ligand. These results are similar to those obtained for third generation Casiopeínas^®^ anticancer copper complexes, which present cytotoxic activity, among others producing reactive oxygen species (ROS) [42].

### 3.4. Lipophilicity

The lipophilicity of the ligands follows the order tetra > batho > bam > 4metil-phen ≈ 5nitro-phen ≈ neo > phen > dmb > bipy (Table S2 in the Supplementary Information). Taking this into account, it is expected that different diimines may be able to regulate the lipophilicity of the ternary complexes. Coordination with the most lipophilic ligands (tmp, batho and bam) produces the most lipophilic complexes. Introducing one or two methyl groups in the phen ligand or a nitro substituent does not produce significant differences in lipophilicity determined by this method, conforming an intermediate group. The most hydrophilic are complexes with bipy, dmb, and phen ligands.

**Figure 4:**
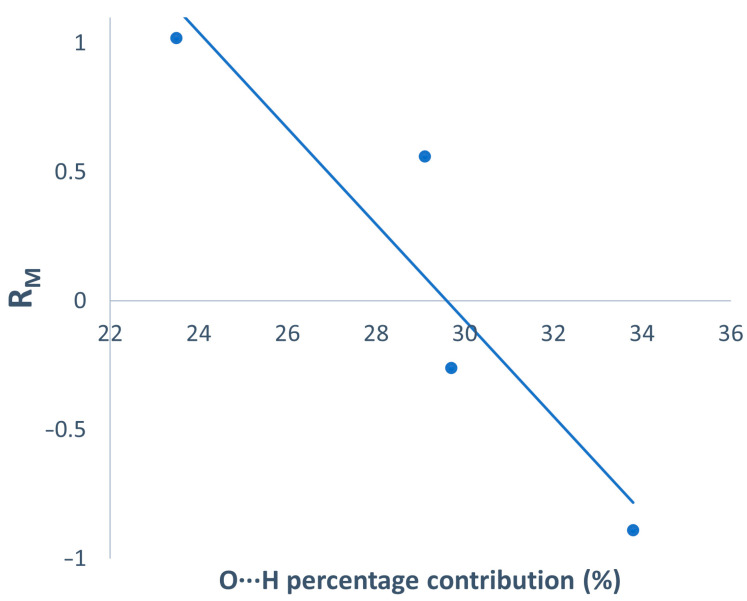
O···H percentage contact contribution to the *d*_norm_ Hirshfeld surface vs. R_M_.

Considering the variability in the lipophilicity, the neutral nature of the aqueous solution major species, and the existing crystal structures, relationships between semiquantitative intermolecular contacts (in the lattice) and lipophilicity were explored. To that end, Hirshfeld surface analysis was performed on data from complexes (**1**), (**4**), (**7**), and (**8**), all of which correspond to structures containing discrete mononuclear units (Hirshfeld surfaces and contact distributions are available in Figures S8 and S9, Table S3). Figure 4 shows the linear dependency of the contribution of the most polar intermolecular contacts in the lattice (mainly O···H contacts from classical H-bonds chosen as a hydrophilicity descriptor) with the determined lipophilicity. The least lipophilic compounds are those which present the highest percentage contributions of O···H contacts to the overall Hirshfeld surface. The percentage distribution of polar contacts in the crystal structure can be considered a lipophilicity descriptor for the studied complexes.

### 3.5. DNA Binding

DNA binding is considered part of the mechanisms of action for copper(II) complexes containing planar heterocyclic ligands [43]. In this work, we aimed to understand the changes produced in the DNA tertiary and secondary structures and the binding strength using three complementary methodologies: Spectroscopical determination of the intrinsic binding constant, circular dichroism spectroscopy, and viscosity measurements.

All recorded spectra and Benesi–Hildebrand linearization curves are available in Figures S10 and S11 in the Supplementary Information.

Table 5 summarizes the results from the determination of *K*_b_ and circular dichroism spectra.

**Figure 5:**
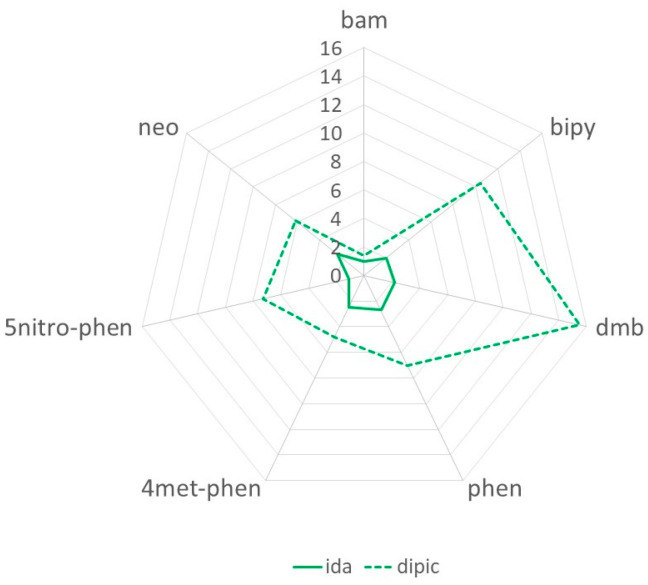
*K*_b_ × 10^3^ values for iminodiacetic (solid line and dipicolinate (dotted line) complexes with diiminic ligands.

Binding constants for [Cu(dipic)(NN)(H_2_O)_x_] are in the range 1 and 16 × 10^3^ M^−1^, presenting similar results to those observed for the ternary copper(II) complex containing dipicolinate and 3-amino-1H-1,2,4-triazole [44]. These values, in most cases, are significantly higher than those determined in the same conditions with the more flexible iminodiacetate ligand [13], indicating the geometrical rigidity of the dipic positively impacts the formation of the complex-DNA adduct (Figure 5). Moreover, dipic complexes present a higher differentiation in the binding strength of the different diiminic ligands, following the order: dmb > bipy >> 5nitro-phen ≈ phen > neo > tmp ≈ 4met-phen ≈ batho > bam. Interestingly, the tendency observed for DNA affinity is the opposite of that observed for lipophilicity (Figure S12, Supplementary Information). The most hydrophilic complexes (bipy, dmb, and phen) present the highest *K*_b_ values, whereas the most lipophilic ones present the lower *K*_b_ values (batho and bam). This trend indicates that an increase in the polarity of the compound enhances DNA affinity.

**Figure 6:**
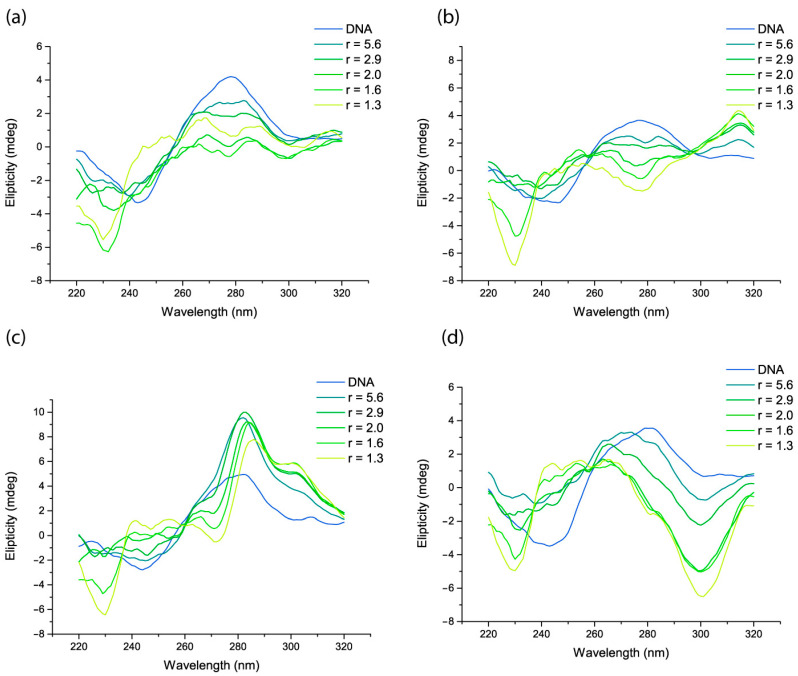
Circular dichroism spectra with increasing [DNA]/[complex] (*r*) ratio for complexes (**1**) (**a**), (**2**) (**b**), (**4**) (**c**) and (**8**) (**d**).

Circular dichroism studies suggest that the conformational changes caused by the complexes depend on the diiminic ligand (Figure 6 and Figure S13 in Supplementary Information).

The observed changes in the characteristic bands can be summarized as follows:

- Decrease in the intensity of the 275 nm band without modification of the 245 nm band. Characteristic of B- to C-form conformational change in the DNA. Observed for complexes (**1**), (**2**), (**6**), and (**7**);
- Simultaneous intensification of the 245 and 275 nm bands with a shift to higher wavelengths in the latter. This is consistent with B- to A-form conformational changes in the DNA. Observed for complexes (**4**), (**5**), and (**9**);
- Inversion of the circular dichroism spectra, in agreement with the conformational change from the right-handed B-form to the left-handed Z-form of the DNA. Observed for complex (**8**).
- Appearance of a positive band around 300 nm suggesting DNA condensation. Observed for complex (**3**).

Compared to related complexes with the Cu-iminodiacetate scaffold, the dipic modifies, in some cases, the conformational changes induced in the DNA, as detected through CD. In the case of complexes (**1**), (**2**), and (**7**) (bam, bipy, and neo), there is a shift from a partial intercalation mode for iminodiacetate to a B- to C-form conformational change for dipic. For other complexes, the induced conformational changes are the same. In the case of complex (**3**) with dmb, both the iminodiacetate and the dipic complexes produce DNA condensation. Complexes (**4**) and (**5**), with phen and 4met-phen, evidence a B- to A-form conformational change, and complex (**6**) with 5nitro-phen suggests a B- to C-form change, being the same profile as its iminodiacetate analog.

**Figure 7:**
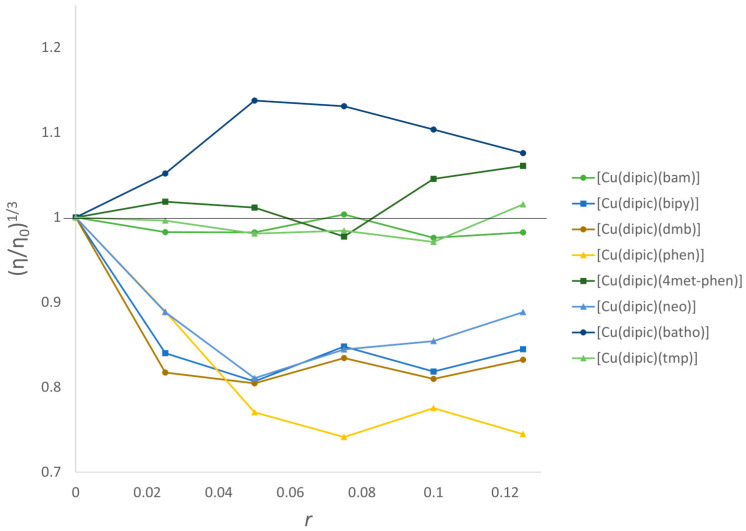
Effect of the increasing concentration of complexes on the relative viscosity of CT-DNA. [DNA] = 150 µM, for [Cu(dipicolinate)(diimine)] complexes.

The relative viscosity of CT-DNA in the presence of complexes (**1**), (**2**), (**3**), (**4**), (**5**), (**8**), and (**9**) was determined (Figure 7).

Relative viscosity is a highly sensitive method to detect changes in the overall length of the DNA caused by the interaction of small molecules [45,46]. Complexes (**1**), (**2**), (**3**), and (**7**) show a viscosity decrease and saturation at r = 0.02 and 0.04. This is consistent with a binding mode that provokes bends or kinks in the DNA helix evidenced as a B- to C-form conformational change in the circular dichroism spectra (or condensation for (**3**)).

Complex (**4**) also presents a marked decrease in viscosity. However, circular dichroism evidences a B- to A-form conformational change in which the axial rise between contiguous base pairs decreases significantly compared to the natural B-form, resulting in a smaller effective length and consequent decrease in viscosity [45].

Complex (**5**) presents a slight increase in viscosity, possibly accounting for partial intercalation or groove binding. Nonetheless, circular dichroism evidences a B- to A-form conformational change. These results cannot be correlated with a particular binding mode, to the best of our knowledge.

Increasing concentrations of complex (**9**) does not induce a significant alteration in DNA viscosity. This behavior is related to the role of the methyl groups in impairing DNA intercalation and in agreement with what has been previously reported by Palaniandavar et al. [47] for related [Cu(diimine)_2_]^2+^ complexes, including Cu-neo as detected by fiber EPR studies [48] and reported for related [Cu(dipeptide)(tmp)] [12].

Concerning complex (**8**), containing batho, viscosity and the spectroscopically determined binding constant results are compatible with DNA intercalation [49]. Despite that, circular dichroism spectra in the presence of increasing amounts of complex evidence of a conformational change from the B- to the Z-form. The main difference between B- and Z-forms is that the helix goes from right- to an elongated left-handed chain [50], explaining the observed increase in relative viscosity.

Summarizing, bipy and dmb bind to the DNA with similar binding constants, circular dichroism, and relative viscosity response to the addition of complex. On the other hand, subtle changes in the phen-based ligand strongly influence the binding constant and the induced conformational changes, as evidenced by circular dichroism and relative viscosity.

### 3.6. Cytotoxic Activity

The complexes were cytotoxic against the studied cell lines, as presented in Table 6. Most complexes, except that of 5nitro-phen (**6**), present higher activity than Cisplatin on the studied cell lines and are potent cytotoxic agents according to the classification of Santini et al. [51] of antitumor copper complexes. Complexes are highly active compared to other ternary complexes containing a phen-based ligand, including Casiopeínas^®^ [42]. As compared with the previously studied [Cu(dipeptide)(diimine)] of the same diimine, the activity changed, evidencing the importance of the scaffold and not only the diimine when comparing different coordination environments [8,9,10,11,12]. Even the cells more sensitive to a diimine were modified by changing the Cu-ligand scaffold.

Although the mechanism of action of copper complexes usually includes DNA binding and ROS production, in this work, no relation was found between the cytotoxicity and DNA binding or E_1/2_.

Complexes are also cytotoxic to non-tumor cells, which is a limitation to the possible use of most of the complexes. Despite that, there is a reasonable selectivity towards a cancer cell line for some complexes. Therefore, complexes containing bam (**1**) and dmb (**3**) are interesting to test on animal models of MDA-MB-231, whereas 4met-phen-containing complex (**5**) is more active on MCF-7 and could be tested in this class of tumors. In addition, complexes are very cytotoxic to the A2780cis (resistant to Cisplatin) cell line. In particular, complexes (**5**) and (**9**) present high cytotoxicity against that cell line while being less toxic to non-tumor cells, therefore, they are interesting to be tested on this class of ovarian tumors.

More research in the field is needed to deepen our understanding of the role of the diimine and the anionic ligand in the complexes Cu-ligand-diimine to find more potent complexes as leaders in developing antitumor drugs.

## 4. Conclusions

Four new copper complexes were obtained, including a new crystalline structure of [Cu_2_(dipic)_2_(tmp)_2_]·7H_2_O. In them, the diimine was coordinated perpendicular to the dipic, with the diimine influencing the coordination polyhedra. The major complex species in solution were hexacoordinated, ternary ones, with dimeric complexes hydrolyzing to monomeric ones.

The complexes bound to DNA via different modes, depending on the diimne. Bipy and dmb presented similar DNA binding, whereas for phen-based ligand, subtle changes in the phen substituent modified the binding constant and the induced conformational changes.

Complexes [Cu(dipic)(diimine)] were potent cytotoxic agents, more active than Cisplatin on the studied cell lines, and even active on cells resistant to Cisplatin. Despite that, complexes were also cytotoxic to non-tumor cells. The complexes containing the diimines bam (**1**) and dmb (**3**), due to their relative selectivity towards MDA-MB-231 cancer cells, are interesting to be tested as anticancer drugs on animal models of triple-negative breast cancer. The 4met-phen-containing complex (**5**) is more active on MCF-7 and could be tested in this class of tumors as well as in ovarian cancer resistant to Cisplatin A2780cis.

The neutral Cu-ligand scaffold influenced the cytotoxicity as well as the diimine, evidencing its importance in the biological activity, as observed comparing the activity of [Cu(dipic)(diimine)] with other [Cu(ligand)(diimine)] complexes of the same diimine.

## Figures and Tables

**Table 1:** Summary of crystal data and structure refinement parameters for (**9**).

Empirical formula	C_46_H_42_Cu_2_N_6_O_10_
Formula weight	965.93
Crystal system/Space group	Monoclinic/C2/c
Unit cell dimensions	*a* = 19.9439(5) Å
	*b* = 15.8648(4) Å
	*c* = 14.8236(3) Å
	β = 92.4110(10)°
Volume	4686.12(19) Å^3^
Z	4
Density (calculated)	1.369 Mg/m^3^
Absorption coefficient	0.969 mm^−1^
F(000)	1992
Crystal size	0.240 × 0.109 × 0.075 mm^3^
Theta range for data collection	2.913–27.137°
Index ranges	−25 ≤ *h* ≤ 25, −20 ≤ *k* ≤ 20, −19 ≤ *l* ≤ 17
Reflections collected	37,337
Independent reflections	5178 [R(int) = 0.0464]
Completeness	99.8%
Max. and min. transmission	0.7455 and 0.6798
Data/restraints/parameters	5178/0/293
Goodness-of-fit on F^2^	1.009
Final R indices [I > 2σ(I)]	R1 = 0.0309, wR2 = 0.0778
R indices (all data)	R1 = 0.0426, wR2 = 0.0831

**Table 2:** Summary of the obtained complexes with crystal structures indicating coordination index, geometry, and equatorial and axial donors.

Complex Code	General Formula	CI ^1^	Geometry ^2^	Equatorial Donors ^3^	Axial Donors ^3^	Ref.
(**1**)	[Cu(dipic)(bam)]·3H_2_O	5	SBP	N_2_O_carb,2_	N_bam_	[28]
(**2**)	[Cu_2_(dipic)_2_(bipy)_2_]·12H_2_O	6	Oh	N_3_O_carb_	O_carb,2_	[29]
(**4**)	[Cu(dipic)(phen)(H_2_O)]·2H_2_O	6	Oh	N_3_O_w_	O_carb,2_	[30]
(**7**)	[Cu(dipic)(neo)]·3H_2_O	5	SBP	N_2_O_carb,2_	N_neo_	[31]
(**8**)	[Cu(dipic)(batho)(H_2_O)]·H_2_O	6	Oh	N_3_O_w_	O_carb,2_	[32]
(**9**)	[Cu_2_(dipic)_2_(tmp)_2_]·7H_2_O	6	Oh	N_3_O_carb_	O_carb,2_	This work

^1^ CI: Coordination number, ^2^ Oh: Octahedral, SBP: Square-based pyramid, ^3^ O_carb_ = carboxylic oxygen, O_w_ = water molecule oxygen.

**Table 3:** Bond lengths and angles around the copper(II) center in the crystal structure of (**9**).

Bond Lengths (Å)		Bond Angles (°)			
Cu1-O1	1.9917(12)	O1-Cu1-N2	88.07(6)	O1-Cu1-O3 ^1^	107.76(6)
Cu1-N2	1.9980(15)	O1-Cu1-N3 ^1^	95.24(5)	N2-Cu1-O3 ^1^	97.32(6)
Cu1-N3 ^1^	2.0215(15)	N2-Cu1-N3 ^1^	175.03(9)	N3′-Cu1-O3 ^1^	78.26(6)
Cu1-N1	2.0470(15)	O1-Cu1-N1	155.35(6)	N1-Cu1-O3 ^1^	95.71(6)
Cu1-O3 ^1^	2.2019(14)	N2-Cu1-N1	81.41(6)		
		N3 ^1^-Cu1-N1	96.86(6)		

^1^ Symmetry related: 1-x, y, ½ -z.

**Table 4:** Maximum absorption wavelength (λ_max_, nm), molar absorptivity (ε, M^−1^ cm^−1^) in aqueous and DMSO solutions for [Cu(dipic)(NN)(H_2_O)_x_] complexes, and E°’_1/2_ NHE.

Complex Code	Complex	λ_max_ (nm)/ε (M^−1^ cm^−1^) H_2_O	λ_max_ (nm)/ε (M^−1^ cm^−1^) DMSO	E°′_1/2_ vs. NHE (mV)
(**1**)	[Cu(dipic)(bam)]	708/16	788/121	ND
(**2**)	[Cu_2_(dipic)_2_(bipy)_2_]	711/42	761/97	−0.058
(**3**)	[Cu(dipic)(dmb)]	722/41	761/104	0.091
(**4**)	[Cu(dipic)(phen)(H_2_O)]	ND	760/87	0.157
(**5**)	[Cu(dipic)(4met-phen)]	730/42	738/72	0.183
(**6**)	[Cu(dipic)(5nitro-phen)(H_2_O)]	715/38	743/108	ND
(**7**)	[Cu(dipic)(neo)]	756/-	746/149	ND
(**8**)	[Cu(dipic)(batho)(H_2_O)]	ND	746/130	ND
(**9**)	[Cu(dipic)(tmp)]	ND	744/96	ND

ND: not determined.

**Table 5:** Summary of DNA interaction findings, intrinsic binding constant, and evidenced conformational change in circular dichroism.

	Compound	*K*_b_ × 10^3^ (M^−1^)	Conformational Change
(**1**)	[Cu(dipic)bam]	1.4	B **→** C
(**2**)	[Cu(dipic)bipy]	10.4	B **→** C
(**3**)	[Cu(dipic)dmb]	15.5	condensation
(**4**)	[Cu(dipic)phen]	7.0	B **→** A
(**5**)	[Cu(dipic)(4metil-phen)]	4.8	B **→** A
(**6**)	[Cu(dipic)(5nitro-phen)]	7.3	B **→** C
(**7**)	[Cu(dipic)neo]	6.2	B **→** C
(**8**)	[Cu(dipic)batho]	4.2	B **→** Z
(**9**)	[Cu(dipic)(tmp)]	4.9	B **→** A

**Table 6:** Cytotoxic activity (expressed by IC_50_ in µM) of the studied complexes after 48 h of incubation against MCF-7, MDA-MB-231 (human breast adenocarcinomas, the latter triple negative), MCF-10A (breast non-tumoral), A549 (human lung epithelial carcinoma), MRC-5 (lung non-tumoral), and A2780cis (human ovarian Cisplatin-resistant).

Complex	MCF-7	MDA-MB-231	MCF-10A	A549	MRC-5	A2780cis
(**1**)	2.18 ± 0.17	0.26 ± 0.02	1.76 ± 0.16	20.47 ± 1.76	3.85 ± 0.08	23.75 ± 8.78
(**3**)	8.83 ± 0.73	0.62 ± 0.07	1.73 ± 0.38	15.06 ± 4.38	1.15 ± 0.10	2.63 ± 0.31
(**4**)	3.70 ± 0.39	3.14 ± 0.45	12.45 ± 1.27	6.16 ± 0.82	1.20 ± 0.19	1.43 ± 0.13
(**5**)	0.58 ± 0.14	2.61 ± 0.15	1.72 ± 0.07	2.00 ± 0.09	5.49 ± 0.61	0.63 ± 0.04
(**6**)	10.35 ± 1.74	16.75 ± 0.35	14.74 ± 0.33	20.06 ± 0.83	11.67 ± 0.90	4.03 ± 0.03
(**7**)	0.34 ± 0.04	7.08 ± 0.46	0.88 ± 0.09	0.26 ± 0.01	0.51± 0.13	0.42 ± 0.02
(**8**)	0.85 ± 0.18	0.38 ± 0.03	0.59 ± 0.10	0.30 ± 0.01	0.05 ± 0.01	0.23 ± 0.002
(**9**)	1.27 ± 0.19	0.73 ± 0.02	1.09 ± 0.06	1.34 ± 0.19	0.41 ± 0.07	0.24 ± 0.05
Cisplatin	8.58 ± 1.77	10.23 ± 0.20	2.91 ±0.17	14.42 ± 1.45	29.09 ± 0.78	26.89 ± 0.60

## Data Availability

The data presented in this study are available in the article and Supplementary Materials.

## Supplementary Materials

The following supporting information can be downloaded at: https://www.mdpi.com/article/10.3390/pharmaceutics15051345/s1, Figure S1. FT-IR of studied [Cu(dipic)(NN)] complexes. Figure S2. Ortep 50% probability representation of the asymmetric unit of [Cu_2_(dipic)_2_(tmp)_2_]. Figure S3. Proper 2-fold rotation axis in the structure of (9). Figure S4. Intramolecular π···π interactions in the dinuclear [Cu_2_(dipic)_2_(tmp)_2_] moiety. Figure S5. (a) Full interaction maps calculated for [Cu_2_(dipic)_2_(tmp)_2_]·8H_2_O. (b) Surrounding molecules superimposed with the calculated map. Color code: H-bond acceptor (blue) and donor (red) propensity, π-stacking propensity (orange). Figure S6. Room temperature EPR spectra of the aqueous solutions of (2) and (9). Figure S7. Electronic spectra for water and DMSO solutions of the studied complexes. Figure S8. Total interactions fingerprint 2D of (a) [Cu(dipic)(bam)]·3H_2_O, (b) [Cu(dipic)(phen)(H_2_O)]·2H_2_O, (c) [Cu(dipic)(neo)]·3H_2_O y (d) [Cu(dipic)(batho)(H_2_O)]·H_2_O. Figure S9. Percentage contact contribution to the Hirshfeld Surface of [Cu(dipic)(NN)] mononuclear solid-state complexes. Figure S10. Electronic spectra resulting from the complex titration with DNA to determine Kb, r indicates [DNA]/[complex] ratio. Figure S11. Benesi–Hildebrand linearization curves. Figure S12. Kb as a function of RM for the studied complexes. Figure S13. DNA Circular dichroism spectra with increasing concentrations of complex, r is defined as [DNA]/[complex]. Figure S14. Cyclic voltammetry at 150 mV/s scan rate of 1 mM aqueous solutions of complexes (2), (3), (4) and (5) in 0.1 M KCl. Table S1. Thermogravimmetric and differential scanning calorimetry results. Table S2. Lipophilicity for diiminic ligands and [Cu(dipic)(NN)(H2O)x] complexes expressed as RM. Table S3. Percentage contact contribution to the Hirshfeld Surface for mononuclear solid-state complexes of [Cu(dipic)(NN)].
