# Supplementary material for: Development of Copper Complexes with Diimines and Dipicolinate as Anticancer Cytotoxic Agents
Source: Pharmaceutics. 2023 Apr 27;15(5):1345. doi: 10.3390/pharmaceutics15051345 (PMC10221202; doi:10.3390/pharmaceutics15051345)
Supplement: Supplementary file 1 [file pharmaceutics-15-01345-s001.zip › pharmaceutics-2252491-supplementary.pdf]

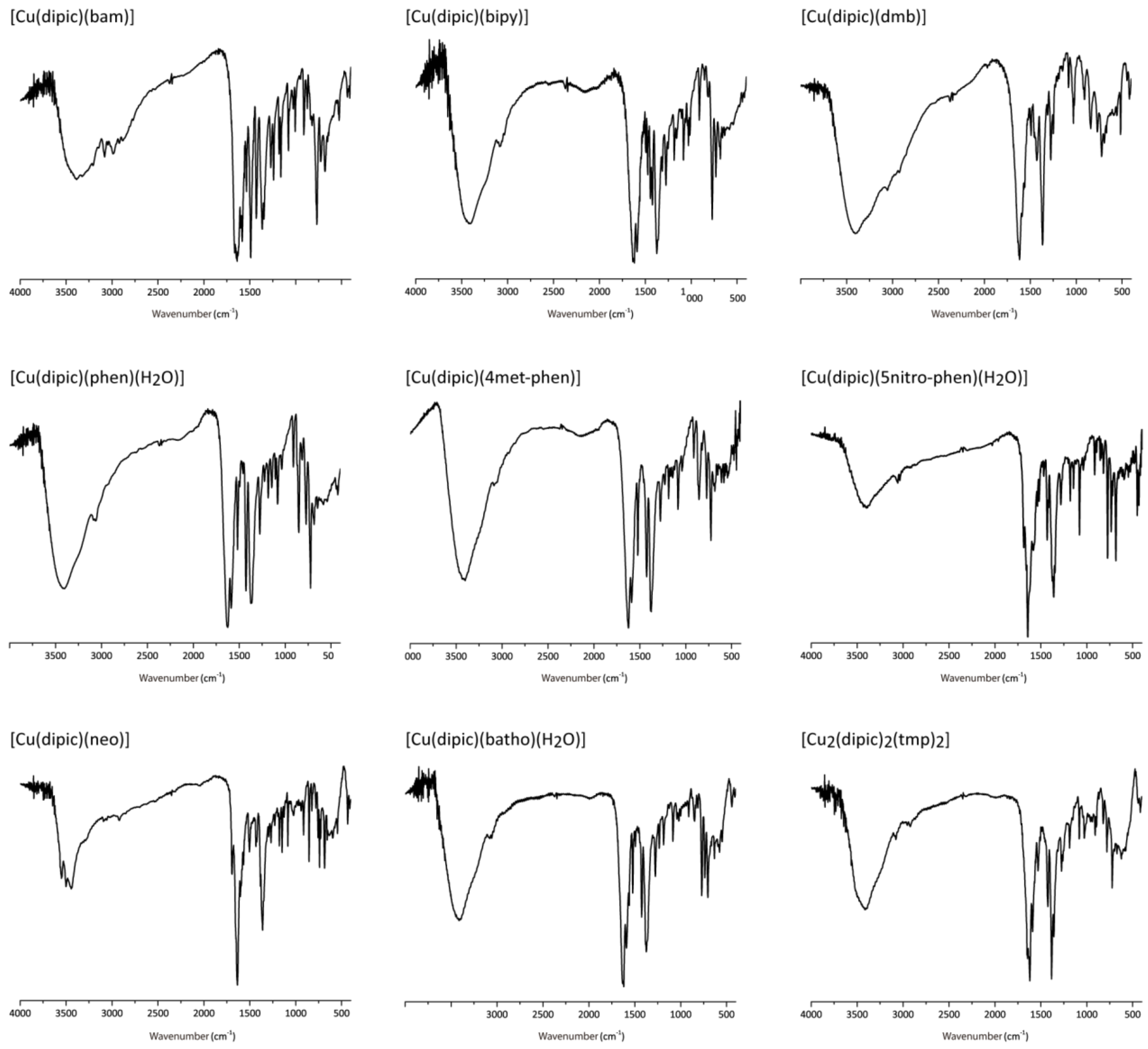

Figure S1. FT-IR of studied [Cu(dipic)(NN)] complexes.

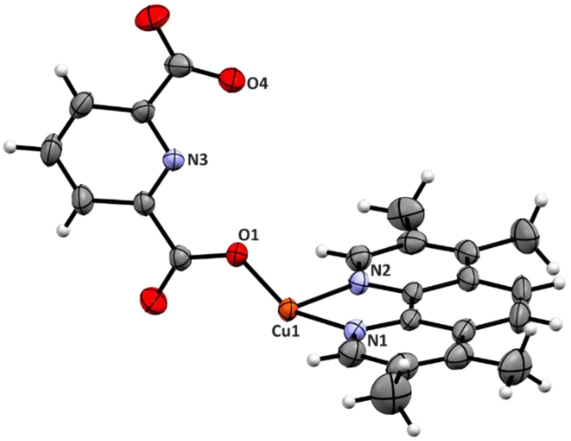

Figure S2. Ortep 50 % probability representation of the assymetric unit of [$Cu_2(dipic)_2(tmp)_2$].

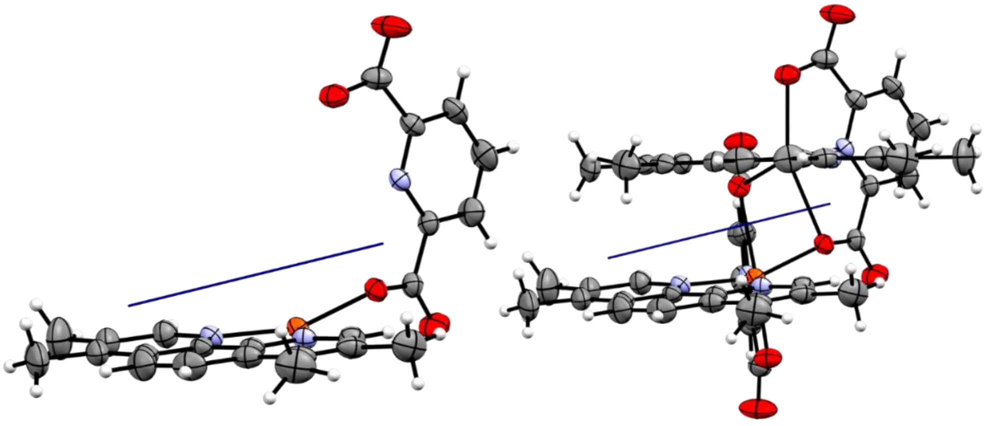

Figure S3. Proper 2-fold rotation axis in the structure of **(9)**.

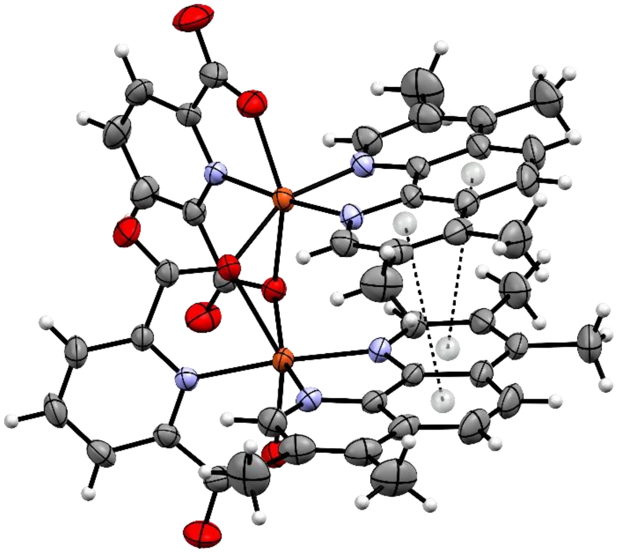

Figure S4. Intramolecular π···π interactions in the dinuclear [$Cu_2(dipic)_2(tmp)_2$] moiety.

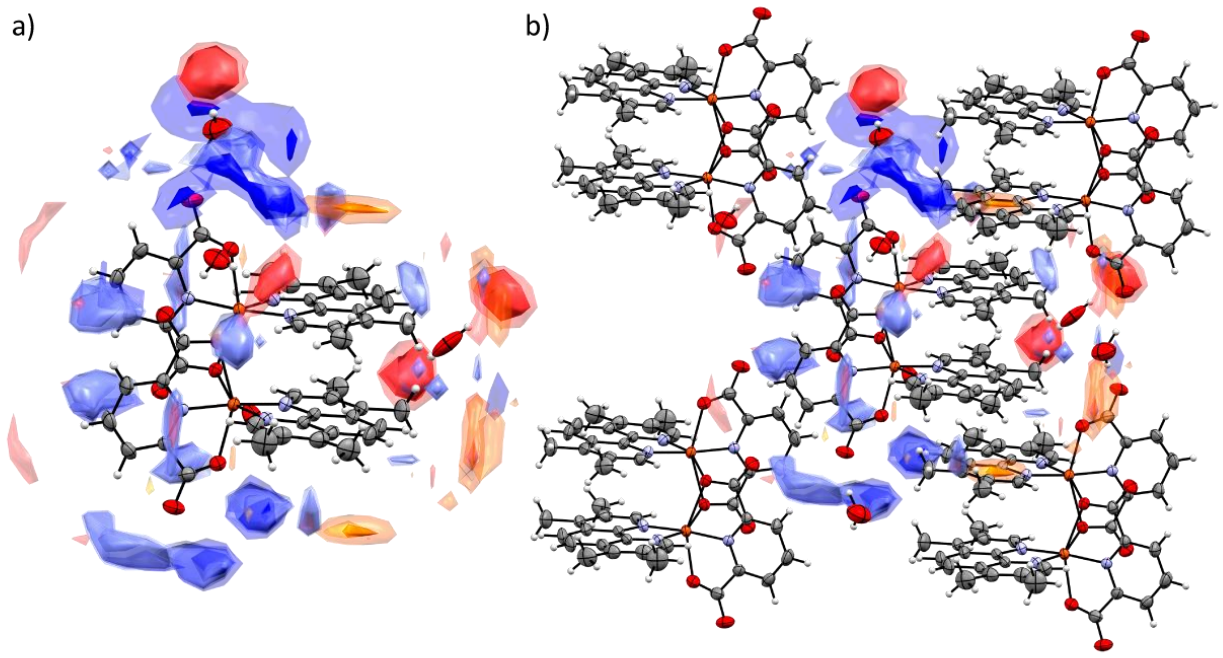

Figure S5. (a) Full interaction maps calculated for $[Cu_2(dipic)_2(tetra\text{-}phen)_2]\cdot 8H_2O$. (b) Surrounding molecules superimposed with the calculated map. Color code: H-bond acceptor (blue) and donor (red) propensity, π-stacking propensity (orange).

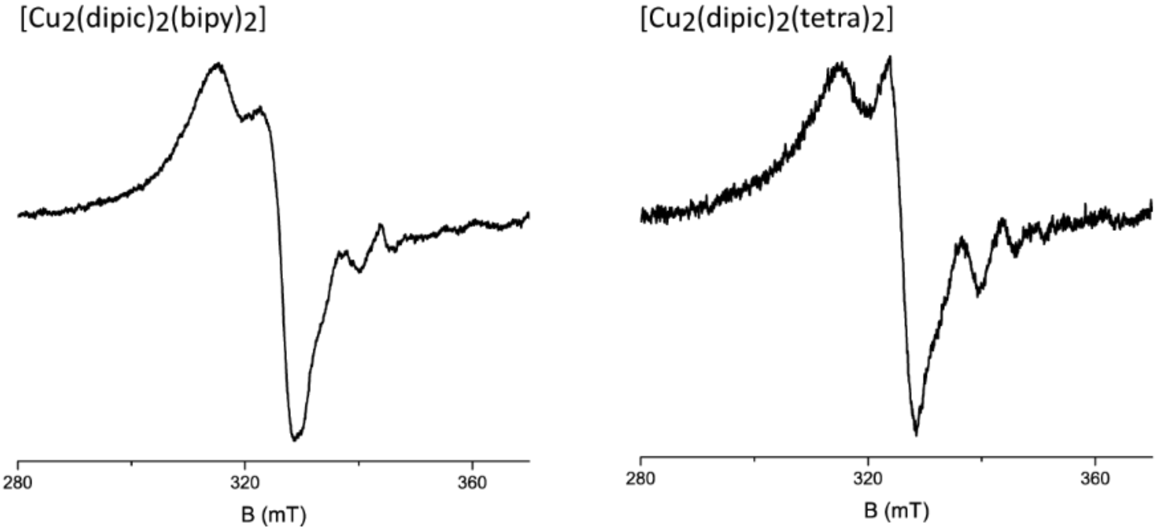

Figure S6. Ambient temperature EPR spectra of the aqueous solutions of **(2)** and **(9)**.

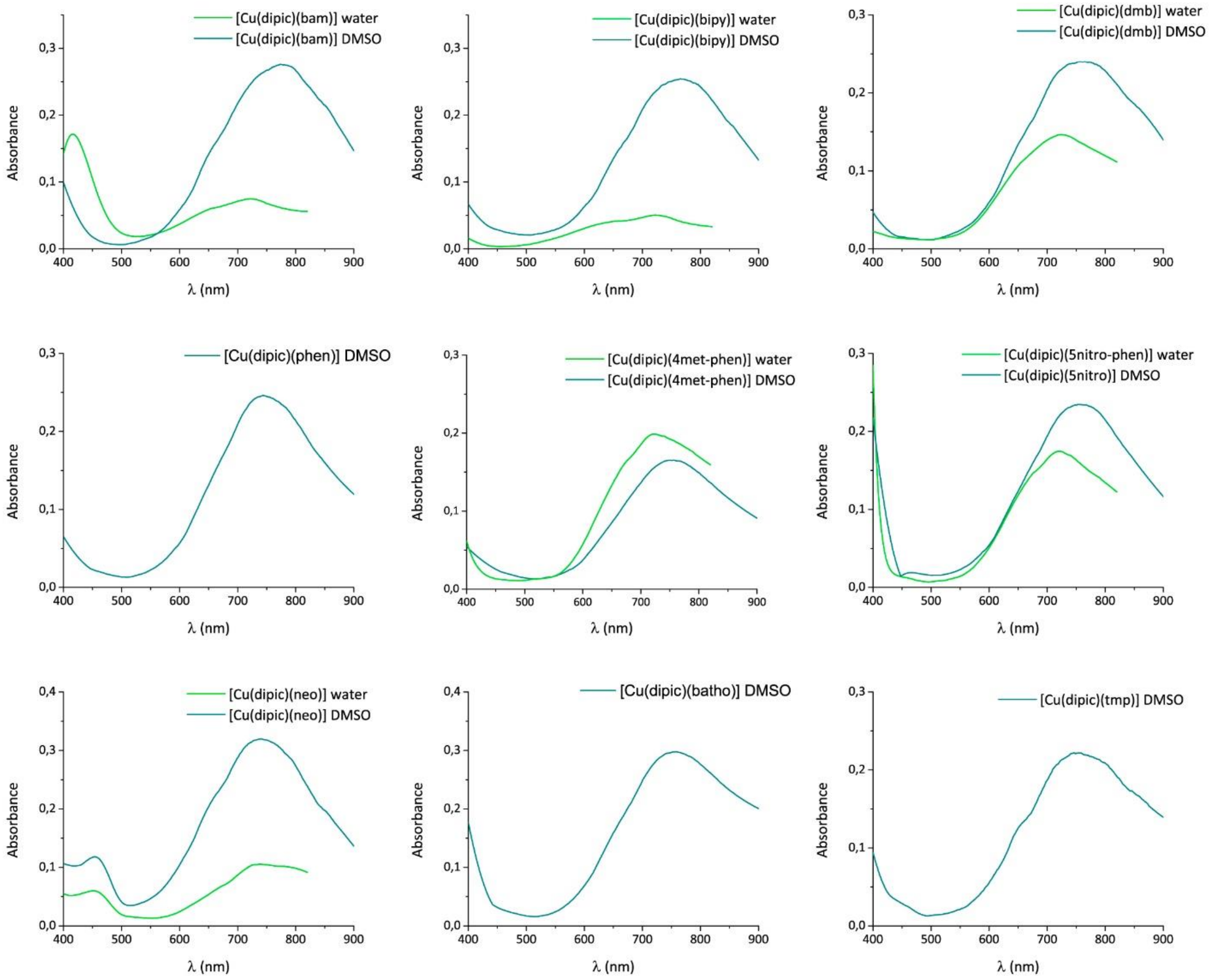

Figure S7. Electronic spectra for water and DMSO solutions of the studied complexes.

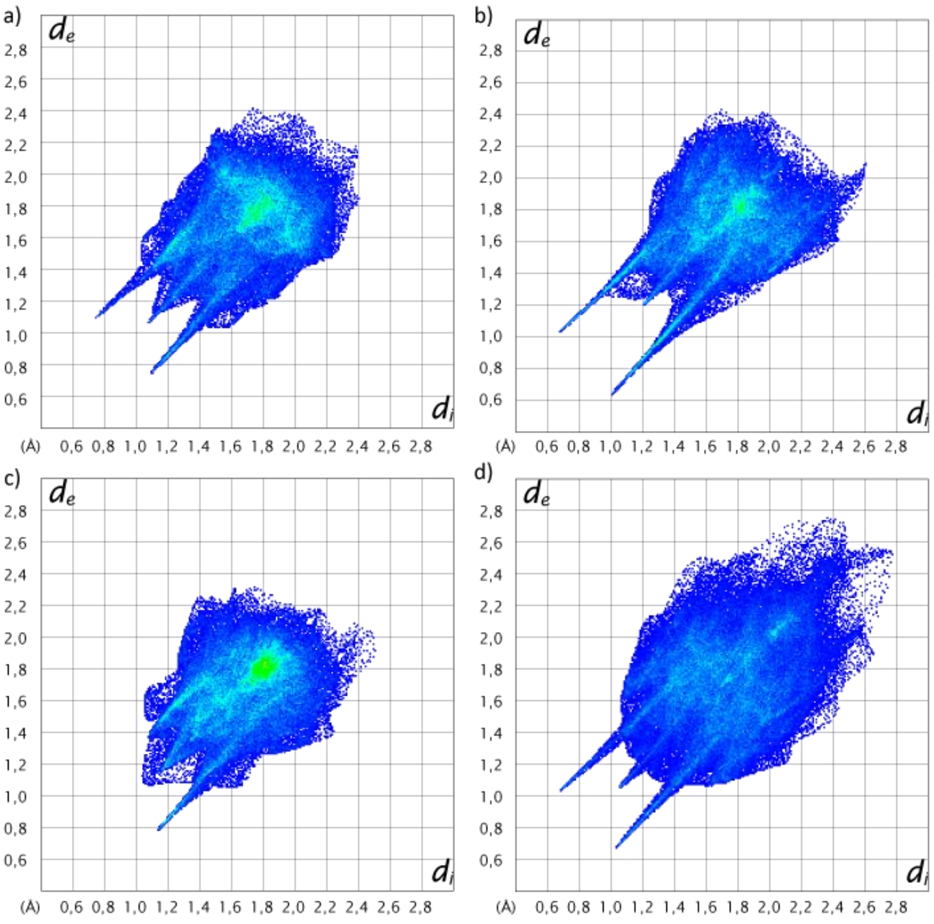

Figure S8. Total interactions fingerprint 2D of (a) [Cu(dipic)(bam)]·3H₂O, (b) [Cu(dipic)(phen)(H₂O)]·2H₂O, (c) [Cu(dipic)(neo)]·3H₂O y (d) [Cu(dipic)(batho)(H₂O)]·H₂O.

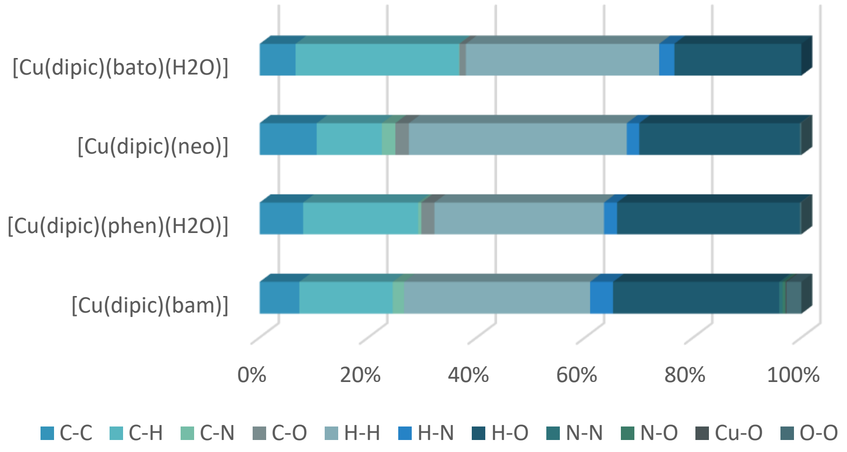

Figure S9. Percentage contact contribution to the Hirshfeld Surface of [Cu(dipic)(NN)] mononuclear solid state complexes.

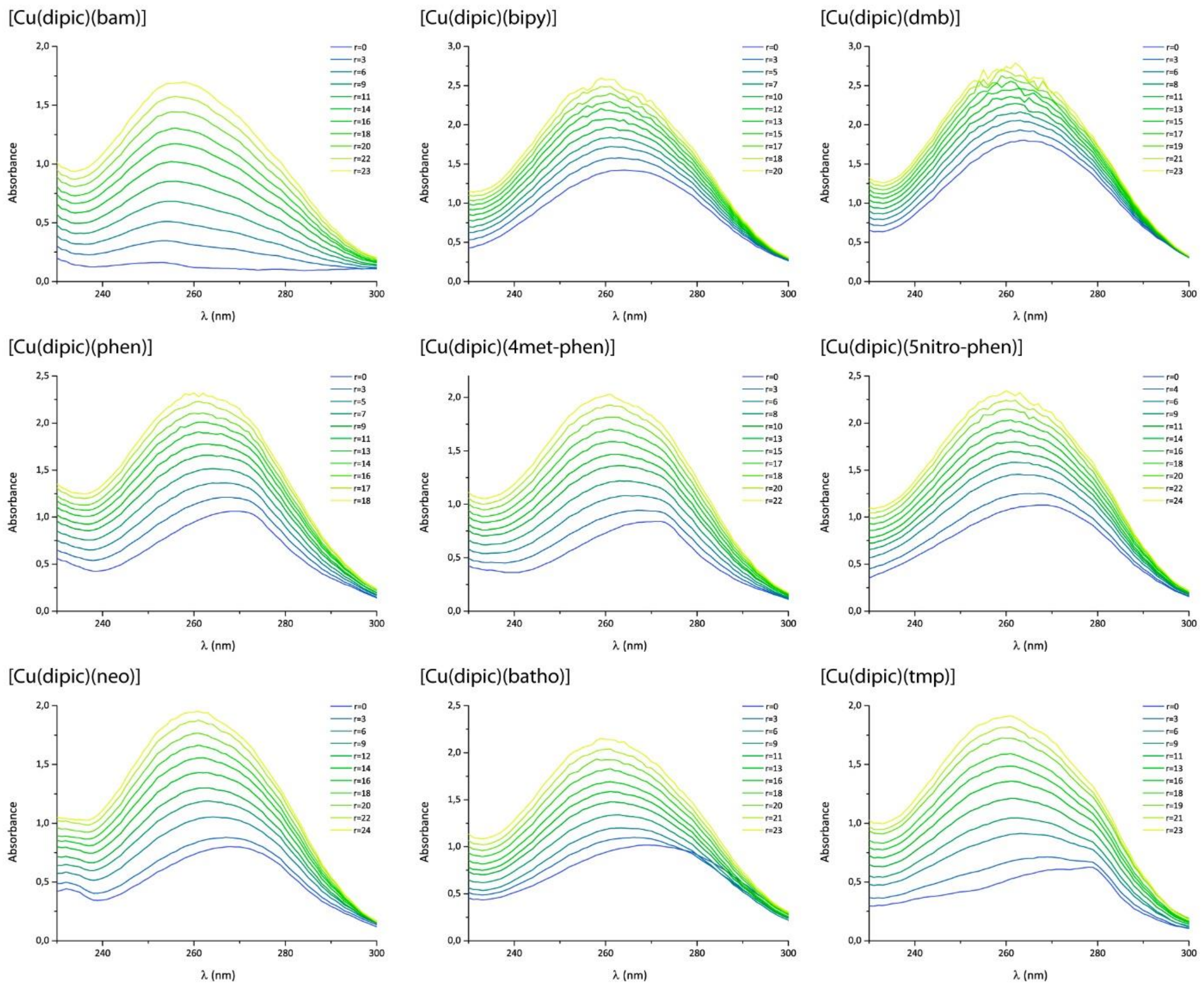

Figure S10. Electronic spectra resulting from the complex titration with DNA to determine K_b, *r* indicates [DNA]/[complex] ratio.

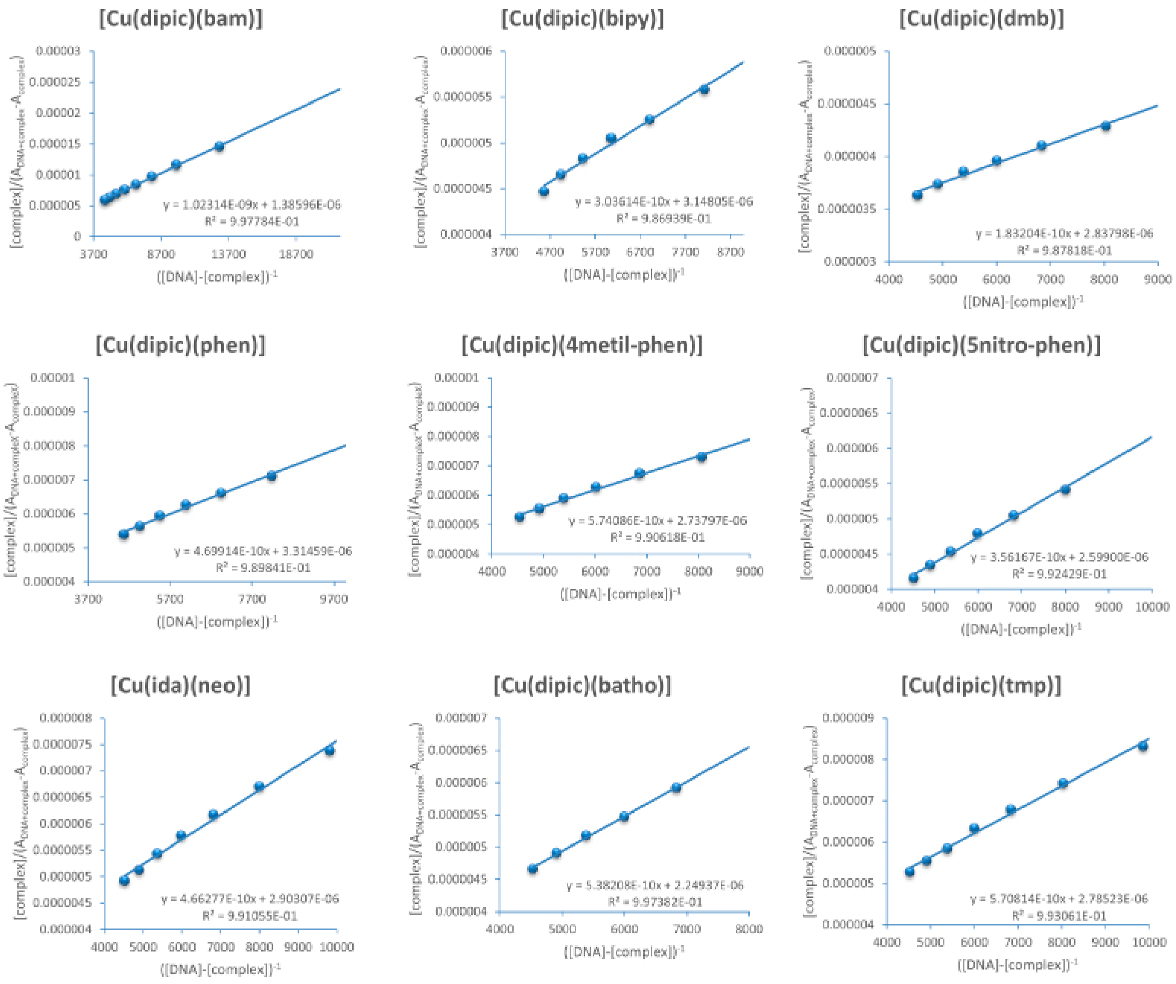

Figure S11. Benesi-Hildebrand linearization curves.

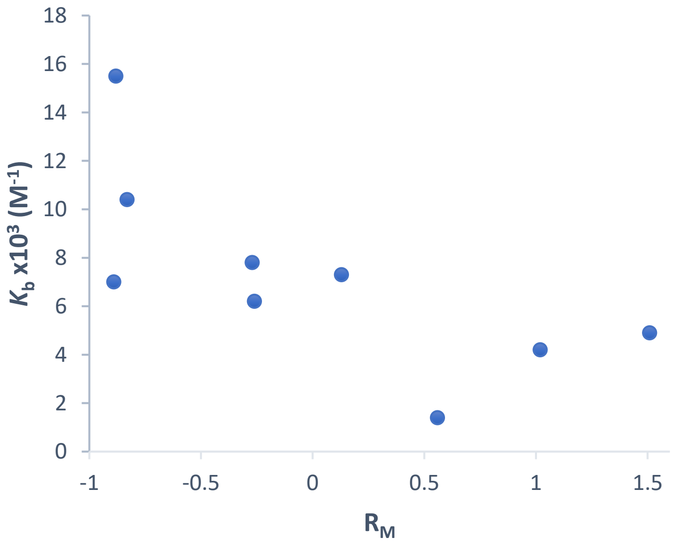

Figure S12. K_b as a function of R_M for the studied complexes.

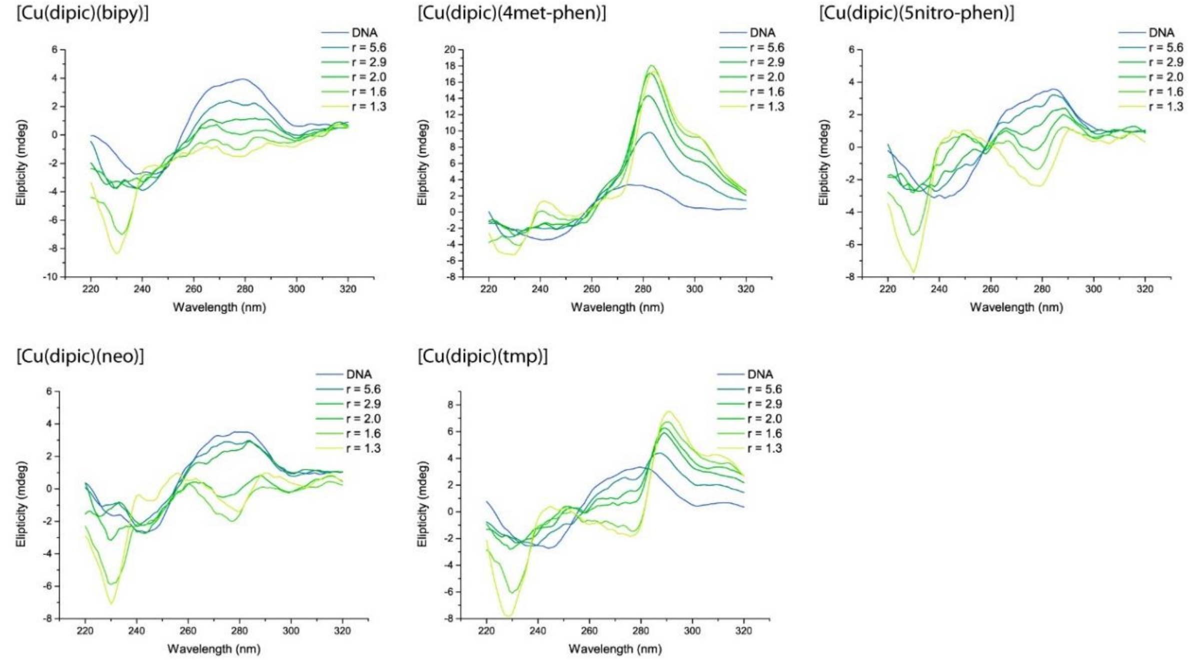

Figure S13. DNA Circular dichroism spectra with increasing concentrations of complex, *r* is defined as [DNA]/[complex].

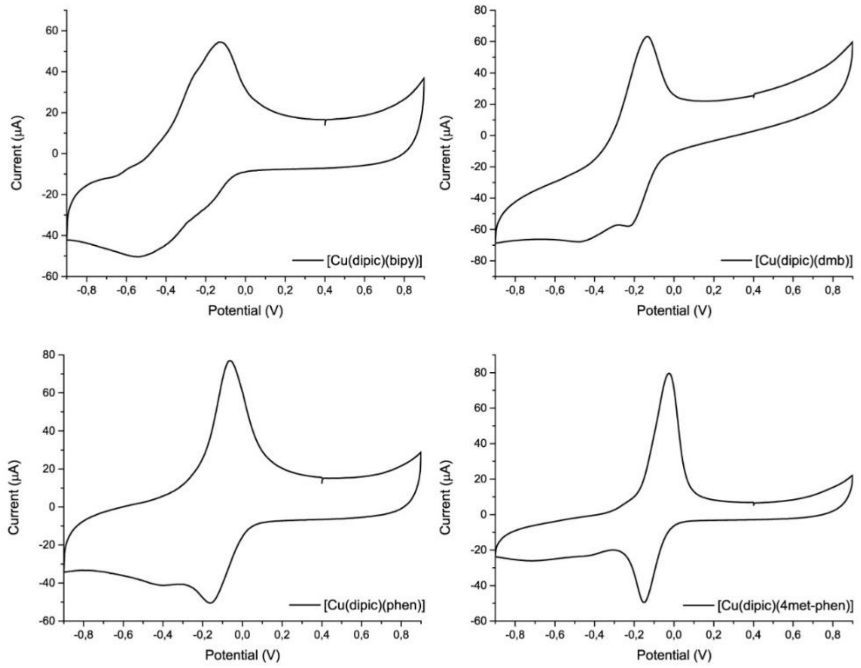

Figure S14. Cyclic voltammetry at 150 mV/s scan rate of 1 mM aqueous solutions of complexes **(2)**, **(3)**, **(4)** and **(5)** in 0.1 M KCl.

Table S1. Thermogravimetric and differential scanning calorimetry results.

Formula	Temp. (°C)	Exp. Weight loss (%)	Theo. Weight loss (%)	Fragment	Δ*H* (kJ/mol)	Comment
**[Cu(dipic)(bam)]·3.5H_2O**	25-268	12.48	11.91	3.5H_2O	149.01 (60 °C)	
	268-800				Melt: 67.58 (269°C)	Incomplete decomposition
**[Cu(dipic)(bipy)]·2.5H_2O**	25-172	10.03	10.48	2.5H_2O	91.55 (98 °C)	
	172-320	56.8	55.67	bipy + dmpy		dmpy = dimethyl-pyridine
	320-800	14.43	16.75	CO_2+CO		Final residue: CuO
**[Cu(dipic)(dmb)]·6.5H_2O**	25-62	7.86	8.5	2.5H2O	38.67 (45 °C)	
	62-138	12.31	13.6	4H2O	83.86 (80 °C)	
	138-344	48.17	45.16	dmb + dmpy	Melt: 77.73 (253 °C)	
	344-800	14.67	13.59	CO_2+CO		Final residue: CuO
**[Cu(dipic)(phen)(H_2O)]·2H_2O**	25-177	11.73	11.68	3H_2O	88.73 (64 °C)	
	177-800	60.89	59.48	phen + dmpy	Melt: 77.73 (253 °C)	Final residue: CuC_2O_4
**[Cu(dipic)(4metil-phen)]·2.5H_2O**	25-187	11.06	11.33	3H2O	127.52 (65 °C)	
	187-800	59.98	60.68	4metil-phen + dmpy		Final residue: CuC_2O_4
**[Cu(dipic)(5nitro-phen)(H_2O)]·2H_2O**	25-190	10.79	10.64	3H_2O	58.99/77.27 (104/158 °C)	Two energetic steps (2H_2O/H_2O)
	190-800	50.58				Incomplete decomposition
**[Cu(dipic)(neo)]·2.5H_2O**	25-97	9.19	9.34	2.5H_2O	103.88 (48 °C)	
	97-345	44.78	43.21	neo		
	345-800	15.20	17.25	dmpy		Final residue: CuC_2O_4
**[Cu(dipic)(batho)(H_2O)]·H_2O**	25-241	5.90	6.03	2H_2O	100.5 (120 °C)	
	241-800	55.37	55.67	batho		[Cu(dipic)]

Table S2. Lipophilicity for diiminic ligands and [Cu(dipic)(NN)(H_2O)$_x$] complexes expressed as R_M.

NN	R_M (ligand)	R_M (complex)
bipy	-0.36	-0.83
dmb	-0.25	-0.88
phen	-0.19	-0.89
neo	-0.14	-0.26
4met-phen	-0.06	-0.27
5nitro-phen	-0.10	0.13
bam	0.20	0.56
batho	0.26	1.02
tmp	0.35	1.51

Table S3. Percentage contact contribution to the Hirshfeld Surface for mononuclear solid-state complexes of [Cu(dipic)(NN)].

	(1)	(4)	(7)	(8)
**C···C**	6.9	8	10.5	6.6
**C···H**	16.3	21.2	12	30.1
**C···N**	2	0.6	2.5	0.1
**C···O**	0	2.4	2.5	1.2
**H···H**	32.5	31.3	40.2	35.7
**H···N**	4	2.4	2.3	2.8
**H···O**	29.1	33.8	29.7	23.5
**N···N**	0.6	0	0	0
**N···O**	0.5	0	0	0
**Cu···O**	0.2	0	0	0
**O···O**	2.6	0.3	0.3	0
